# The gut microbiome mediates the association between a flavonoid-rich diet and MASLD in a population-level analysis

**DOI:** 10.1007/s00394-025-03851-2

**Published:** 2025-12-04

**Authors:** William Bell, Amy Jennings, Nicola P. Bondonno, Andre Franke, Corinna Bang, Marcus Both, Jan Kassubek, Hans-Peter Müller, Jan Borggrefe, Wolfgang Lieb, Tilman Kühn, Aedín Cassidy

**Affiliations:** 1 Co-Centre for Sustainable Food Systems and Institute for Global Food Security, Queen’s University Belfast, 19 Chlorine Gardens, Belfast, BT9 5DL Northern Ireland, UK; 2 Department of Nutritional Sciences, University of Vienna, Vienna, Austria; 3 Center for Public Health, Medical University of Vienna, Vienna, Austria; 4 Danish Cancer Institute, Copenhagen, Denmark; 5 Nutrition and Health Innovation Research Institute, School of Medical and Health Sciences, Edith Cowan University, Joondalup, WA Australia; 6 Institute of Clinical Molecular Biology, University Hospital Schleswig-Holstein, Campus Kiel and Kiel University, Kiel, Germany; 7 Department of Radiology and Neuroradiology, University Hospital Schleswig-Holstein, Campus Kiel and Kiel University, Kiel, Germany; 8 Department of Neurology, University of Ulm, Ulm, Germany; 9 Department of Radiology, Mühlenkreiskliniken Minden, Ruhr-University Bochum, Bochum, Germany; 10 Institute of Epidemiology and Biobank PopGen, University Hospital Schleswig-Holstein, Campus Kiel and Kiel University, Kiel, Germany; 11 Cancer Epidemiology Unit, Nuffield Department of Population Health, University of Oxford, Oxford, UK; 12 Department for Nutritional, Food and Consumer Studies, Hochschule Fulda, University of Applied Sciences, Fulda, Germany

**Keywords:** Flavonoids, Microbiome, Mediation, Liver, Nutrition, NAFLD

## Abstract

**Purpose:**

A growing body of evidence suggests diets rich in flavonoids may protect against metabolic-dysfunction associated steatotic liver disease (MASLD) development and progression. As the gut microbiome is important in the biotransformation of flavonoids to their constituent bioactive metabolites, studies on the potential mediating role of the gut microbiome in the association between dietary flavonoid intakes and MASLD are warranted but lacking. Thus, this study aims to examine the associations between a diet rich in flavonoids and MASLD, and assess the potential mediating role of the gut microbiome.

**Methods:**

In a cross-sectional analysis (n = 531), using the FlavoDiet score (FDS), we assessed the association between a flavonoid-rich diet and MASLD (ascertained by magnetic resonance imaging) using multivariable logistic and linear regression. Additionally, we used mediation analysis to identify and assess potential 16S-derived gut microbiome mediators.

**Results:**

Each doubling of the FDS was associated with a 27% lower odds of MASLD (OR: 0.73 [95% CI 0.54–0.98], *p* = 0.04) after multivariable adjustment. 9.2% of this association was mediated by a greater abundance of the genus *Eisenbergiella* (indirect effect ß = − 0.006 [95% CI − 0.019, to − 0.000], *p* = 0.04).

**Conclusion:**

These findings suggest that a flavonoid-rich diet is associated with better liver health, and that the abundance of the *Eisenbergiella* taxa may in part explain the association between a flavonoid-rich diet and MASLD.

**Supplementary Information:**

The online version contains supplementary material available at 10.1007/s00394-025-03851-2.

## Introduction

Metabolic-dysfunction associated steatotic liver disease (MASLD), formerly referred to as non-alcoholic fatty liver disease (NAFLD) [1], is a heterogeneous condition defined as > 5% lipid accumulation in the liver associated with metabolic dysfunction [2, 3]. The following text will use MASLD nomenclature to describe previous literature. MASLD is a progressive condition that develops through a combination of metabolic, lifestyle, and genetic risk factors such as obesity, sedentary lifestyle, and genetic polymorphisms [4] and is estimated to affect approximately 32% of the global population [5]. The clinical manifestation covers a spectrum of severity from steatosis (increased lipid accumulation), which may progress to fibrosis and cirrhosis of hepatic tissue in severe cases [4]. At advanced stages, hepatocyte dysfunction results in metabolic perturbations that drive systemic changes in metabolism and body composition such as impairment of gluconeogenesis and peripheral muscle wasting, predisposing individuals to the development of other conditions such as sarcopenia and frailty, liver failure, hepatocellular carcinoma, and lowered quality of life [4, 6].

Plant-rich dietary patterns have been associated with a lower risk of developing MASLD [7, 8]. One reason for this may be their greater inclusion of plant foods rich in flavonoids, a class of polyphenols that have been shown to improve cardiometabolic health and reduce the risk of subsequent disease [9–15]. Additionally, studies suggest that the health benefits of flavonoids are dependent on our gut microbiome, which interacts with flavonoid compounds to modify their bioavailability and biological activity, with subsequent impacts on health [16]. We have recently demonstrated in a large-scale prospective cohort study in the UK Biobank, that a higher FlavoDiet Score (FDS), indicating higher intakes of flavonoid-rich foods, was associated with a 19% lower risk of MASLD over follow-up, and with lower magnetic resonance imaging (MRI) -derived biomarkers of steatosis [18]. Additionally, a limited number of smaller prospective cohort and cross-sectional studies have reported greater dietary intakes of flavonoid subclasses, such as flavan-3-ols, and flavanones, are associated with lower MASLD risk [19–21]. Furthermore, small-scale intervention trials have shown promise for flavonoid supplementation, including anthocyanins in improving intermediate biomarkers of liver health [22]. However, studies on the potential mediating role of the gut microbiome underlying proposed associations between flavonoid-rich diets and MASLD have not been previously conducted.

Therefore, we aimed to (i) assess the associations between a greater intake of a flavonoid-rich diet [10, 18, 23–25], flavonoid-rich foods, and flavonoid subclasses and prevalent MASLD in a general population sample, and (ii) assess the potential mediating role of the gut microbiome.

## Methods

### Study sample

The Population-Based Recruitment for Genetics Research (PopGen) is a prospective cohort study from Kiel, Germany. From 2005 to 2007, 1317 participants were recruited from population registries (n = 747), and blood donors (n = 569) and attended a standardized examination at the University Hospital in Kiel, where genetic, sociodemographic, biochemical and dietary data were collected. A total of 929 participants were re-examined in 2010–2012 (second examination cycle), when updated data and biological samples were collected and additional measures taken, including stool samples, and a MRI examination of the liver and the subcutaneous and visceral adipose tissues [26–28]. The study was approved by the Christian-Albrechts University of Kiel Ethical review board, and all subjects provided informed written consent.

The present cross-sectional analysis uses data from the second examination cycle (conducted between 2010 and 2012). Exclusion criteria for the present study were as follows: implausible energy intakes (< 600 kcal/day or > 6000 kcal/day), heavy alcohol intake (> 60 g/day for men and > 50 g/day for women) [29], an aspartate transaminase: alanine amino-transferase ratio > 2 which has been shown to identify alcoholic liver disease [30], and other prevalent liver disease diagnosis that may drive steatosis (infectious hepatitis A, B, C, or D, autoimmune liver disease, liver cirrhosis, and hemochromatosis). A flow chart can be found in Supplementary Fig. S1.

### Dietary assessment

All dietary intake data was collected from a self-administered 112-item food frequency questionnaire (FFQ), originally used in the German European Prospective Investigation into Nutrition and Cancer (EPIC) study [31]. Intakes of energy and other nutrients were calculated using the German Food Code and Nutrient Database (version II.3) [17].The primary exposure was our recently reported FDS [10, 18, 23–25]. The FDS is an additive score, comprised of summing the top foods contributing to flavonoid intake in a population, and has been previously applied FDS in American and UK cohorts [10, 25]. Given that sources of flavonoids may vary across populations, we calculated the FDS for the PopGen based on the same number of frequently consumed flavonoid-rich foods in this particular cohort. In PopGen, the FDS is comprised of apples and pears, grapes, red wine, tea, berries, oranges, mandarins and kiwis, and sweet peppers, and food components of the FDS were reported in servings per day. Flavonoid subclass intakes were calculated by assigning flavonoid values to the food items in the FFQ using data from the US Department of Agriculture flavonoid database [32–34], and multiplying these values by the dietary intake data to derive daily estimated flavonoid intakes for each participant. Where values were not available, Phenol Explorer was used to ascertain flavonoid values for the given foods [35]. The calculation of flavonoid intakes in this cohort have also been described elsewhere [36].

### Outcome ascertainment

Liver fat was ascertained via relative liver signal intensity difference and measured using MRI on a 1.5-T Whole-body imager (Magnetom Avanto; Siemens Medical Solutions). Further details on the MRI liver measurement methods including the calculation of liver fat from signal intensity measurements can be found in Koch et al. [28]. Previous analysis on this cohort has used a log liver signal intensity loss of ≥ 3.0 to define steatotic liver, approximating a liver fat of ≥ 5.56% when compared to spectroscopic determined liver fat using receiver operating characteristic analysis as a reference method [28]. In the present study, MASLD was defined as a log liver signal intensity loss of ≥ 3.0 (approximately ≥ 5.56% liver fat) in the absence of both, a heavy alcohol intake (defined as > 60 g/day for men and > 50 g/day for women [29]) and an aspartate transaminase: alanine amino-transferase ratio of less than 2 [30]. The liver enzymes aspartate transaminase and alanine aminotransferase were ascertained via fasting blood samples obtained in a sitting position, and analyzed according to recommendations of the International Federation of Clinical Chemistry. Specifically, concentrations of liver enzymes were ascertained using enzymatic colour tests and photometric detection (Roche Diagnostics). Details on the measurement and sample collection are also described elsewhere [27].

### Assessment of covariates

Covariates were selected via literature search and were as follows: age, sex, education, smoking status, physical activity, energy intake, soluble fibre intake, alcohol intake, BMI, and total coffee intake. Age, sex, education, physical activity, and smoking status were derived from questionnaire data. Nutrient information was derived from the EPIC FFQ as described above under ‘Dietary Assessment’. Height and weight were obtained from physical assessments carried out at the study centre, from which BMI was derived [27]. Details on the study protocols and covariate data collection have also been described elsewhere [27], and coding of each covariate can be found in Supplementary Table S1.

### 16S Microbiome data

16S microbiome data was obtained from faecal samples and extracted via a QIAamp DNA Stool Mini kit on a QIAcube system. MiSeq was then used to sequence the V1-V2 region of the 16S ribosomal RNA gene using the 27F-338R primer pair and dual multiple identifier indexing with the MiSeq Reagent kit (version 3) [37]. MiSeq fastq files were then obtained from base calls for reads 1 and 2, and indexes 1 and 2 using the Bcl2fastq module in CASAVA 1.8.2. No mismatches were allowed in either sequence index. Flash software (version 1.2) [38] was used to merge forward and reverse reads, high quality data derived (sequences with < 5% nucleotides with a quality score > 30 performed with the fastx toolkit), and chimeras removed using UCHIME (version 6) [39]. 10,000 reads for each sample were randomly selected, and sequences clustered at each taxonomic level using the Ribosomal Database Project classifier (reference database version 14) [40]. Classifications with low confidence at the genus level (< 0.8) were organized in an arbitrary taxon of “unclassified family” and Genus-level operational taxonomic units (97% similarity) were created using the UPARSE routine [41].

### Statistical analysis

#### Association of flavonoid intake with MASLD

To analyse the association between flavonoid intakes and the odds of MASLD, we utilized multivariable logistic regression models. Model 1 was adjusted for sex (categorical: male, premenopausal female, postmenopausal female), and age (continuous: years) only. Model 2 was additionally adjusted for education (categorical: low, medium, high), smoking status (categorical: never, under 3 months, previously, current, missing), physical activity (low, medium, high), energy intake (continuous, KJ/day), soluble fibre intake (continuous: g/day), alcohol intake (categorical, < 8 g/day, 8-16 g/day, > 16 g/day), BMI (categorical: ≤ 25 kg/m^2^, > 25 kg/m^2^ and < 30 kg/m^2^, ≥ 30 kg/m^2^ and < 35 kg/m^2^, ≥ 35 kg/m^2^), and total coffee intake (categorical: low, medium, high). Intakes of the FDS and flavonoid subclasses were analysed as continuous variables to maximize power due to the moderate sample size. The individual flavonoid-rich foods were analysed as categorical variables defined by 3 categories due to the distribution of intakes. Intakes defining the categories can be found in Supplementary Table S2 and were chosen based on the distribution of the intake data to maximize the number of participants per group, but approximate: zero intake, < median, and > median intake. In sensitivity analyses, we analysed the association between the FDS and MASLD and between the flavonoid subclasses and MASLD by modelling exposures as quartiles to assess the robustness of our findings. In this analysis, statistical significance was assessed via linear trend test, modeling quartiles as a continuous variable. Mean (SD) flavonoid intakes across quartiles of the FDS can be found in Supplementary Table S3. In all analysis, energy intake, and soluble fiber intake were log transformed, while the FDS and the flavonoid subclasses were log2 transformed to approximate a normal distribution for analysis and improve the interpretability of the score such that the interpretation of the regression coefficient is the difference in MASLD odds per doubling of the variable. Lastly, to test for potential subgroup interactions, we used likelihood ratio testing.

#### Assessing the potential mediating role of the gut microbiome

Due to the high dimensionality of microbiome data, we first removed all microbial taxa with > 60% zero values as previously described in Jennings et al. [36]. We then assessed potential candidates for mediation as per the criteria for mediation analysis described by Baron and Kenny [42]. Using linear regression, we first regressed the microbiome variables on the FDS, selecting those microbiome variables in which the FDS displayed a significant association with. We then used logistic regression to assess which microbiome variables were significantly associated with MASLD status in the cohort. Candidates for mediation were those microbes that were statistically significant in both analyses. Additionally, we assessed potential mediation by alpha diversity measures (Shannon and Simpson indices). Mediation analysis was conducted using the ‘mediation’ package in R [43] and all analyses were bootstrapped for 1000 iterations. All microbiome variables were log-transformed to improve distribution for analysis in regression models.

Lastly, we carried out a leave-one-out sensitivity analysis where we assessed the association between the FDS and MASLD including mediation analysis, excluding each food component to test the robustness of the additive FDS. All tests were considered statistically significant with a two-sided *P* value of < 0.05 All analyses were performed in R version 4.0.2 (R Foundation for Statistical Computing, Vienna, Austria) [44].

## Results

### Sample characteristics

Characteristics of the study sample are presented in Table 1. 531 participants were included in this cross-sectional analysis, of which 201 were classified as having MASLD. The mean (SD) age of the cohort was 60.7 (± 11.9) years, 38.8% were female (of which 27.7% were postmenopausal), and the mean (± SD) BMI was 27.0 kg/m^2^ (± 4.27). Those with MASLD were more likely to be older, overweight, male, and have lower educational attainment. Additionally, those with MASLD were more likely to have smoked, report lower alcohol intakes, and have a lower FDS.

**Table 1:** Characteristics of the Study Sample by MASLD Status

	No MASLD	MASLD	Overall
n	330	201	531
*Sex n (%)*			
Male	196 (59.4)	129 (64.2)	325 (61.2)
Premenopausal female	45 (13.6)	14 (7.0)	59 (11.1)
Postmenopausal female	89 (27.0)	58 (28.9)	147 (27.7)
BMI (kg/m^2^)	25.84 (3.38)	28.89 (4.86)	26.99 (4.27)
Age (years)	58.82 (12.55)	63.89 (10.01)	60.74 (11.90)
*Education Level n (%)*			
None, Primary or middle	89 (27.0)	72 (35.8)	161 (30.3)
Secondary	113 (34.2)	67 (33.3)	180 (33.9)
College and higher education	128 (38.8)	62 (30.8)	190 (35.8)
*Smoking status (%)*			
Never	132 (40.0)	65 (32.3)	197 (37.1)
Less than 3 months	21 (6.4)	16 (8.0)	37 (7.0)
Previously	133 (40.3)	96 (47.8)	229 (43.1)
Current	37 (11.2)	20 (10.0)	57 (10.7)
Missing	7 (2.1)	4 (2.0)	11 (2.1)
*Physical activity (%)*			
Low	102 (30.9)	69 (34.3)	171 (32.2)
Moderate	112 (33.9)	68 (33.8)	180 (33.9)
High	116 (35.1)	64 (31.8)	180 (33.9)
Energy (KJ/Day)	9639 (2825)	9451 (2626)	9568 (2750)
Soluble Fibre (g/day)	0.89 (0.36)	0.87 (0.35)	0.88 (0.36)
Alcohol Intake (g/day)	14.1 (15.1)	12.6 (12.2)	13.5 (14.1)
FDS (servings/day)	3.4 (2.7)	3.0 (2.2)	3.2 (2.5)

*FDS* FlavoDiet Score, *BMI* Body mass index, *MASLD* Metabolic-dysfunction associated steatotic liver disease

Values are mean (± SD)

### Associations of the FDS with MASLD

In the multivariable-adjusted model, each doubling of the FDS was associated with a 27% lower odds of having MASLD (OR [95% CI]: 0.73 [0.54 to 0.98], *p* = 0.04) (Table 2).

**Table 2:** Odds ratio and 95% confidence intervals for the association between the Flavodiet Score, lavonoid Subclasses, and MASLD

Exposure	Model 1		Model 2	
	OR (95% CI)	*P* value	OR (95% CI)	*P* value
*Diet score*				
FDS	0.72 (0.56–0.92)	< 0.01	0.73 (0.54–0.98)	0.04*
*Subclasses*				
Anthocyanins	0.89 (0.75–1.06)	0.19	0.89 (0.72–1.10)	0.30
Proanthocyanidins	0.87 (0.73–1.04)	0.13	0.91 (0.73–1.14)	0.41
Polymers	0.87 (0.72–1.04)	0.13	0.9 (0.72–1.13)	0.36
Flavonols	0.90 (0.73–1.11)	0.31	0.98 (0.76–1.26)	0.86
Flavanones	0.88 (0.77–1.01)	0.06	0.86 (0.74–1.01)	0.06
Flavan-3-ols	0.94 (0.84–1.04)	0.23	0.97 (0.86–1.11)	0.67
Flavones	1.03 (0.81–1.32)	0.81	1.07 (0.80–1.43)	0.65

n = 531, values are OR (95% CI)

Model 1 adjusted for age (years) and sex (male, premenopausal female, postmenopausal female)

Model 2 adjusted for model 1 plus BMI (≤ 25 kg/m^2^, > 25 kg/m^2^ and < 30 kg/m^2^, ≥ 30 kg/m^2^ and < 35 kg/m^2^, ≥ 35 kg/m^2^), education level (none, primary or middle; secondary; higher education), smoking status (never, < 3 months, previous, current, missing), physical activity (low, moderate, high), energy intake (KJ/day), soluble fibre intake (g/day), alcohol intake (< 8 g/day, 8–16 g/day, > 16 g/day), and coffee intake (low, moderate, high)

### Associations between flavonoid-subclasses and MASLD

No statistically significant associations were observed between individual flavonoid subclasses (anthocyanins, proanthocyanidins, polymers, flavones, flavanones, flavan-3-ols, flavonol) and MASLD (OR [95% CI], *P value: anthocyanins = 0.89 [0.72, 1.10], 0.30; proanthocyanidins = 0.91 [0.73, 1.14], 0.41; polymers = 0.90 [0.72, 1.13], 0.36; flavones =* 1.07 [0.80, 1.43], 0.65; flavanones = 0.86 [0.74, 1.01], 0.06; flavan-3-ols = 0.97 [0.86, 1.11], 0.67; flavonols = 0.98 [0.76, 1.26], 0.86) (Table 2).

### Associations between flavonoid-rich foods and MASLD

Compared to the reference low intake, a higher intake of orange, mandarin, and kiwi intake was associated with 49% lower odds of having MASLD in the multivariable-adjusted model (OR (95% CI) T3 vs T1, *P* value: 0.51 (0.31, 0.85), 0.01). No statistically significant associations were observed for other flavonoid-rich foods and MASLD (OR T3 vs T1 [95% CI], P value: apples and pears = 0.72 [0.41, 1.27], 0.39; grapes = 0.92 [0.54, 1.57], 0.84; bell pepper = 1.06 [0.65, 1.73], 0.71; red wine = 0.70 [0.39, 1.25], 0.27; tea = 1.12 [0.72, 1.75], 0.61; berries = 0.95 [0.53, 1.69], 0.75) (Table 3).

**Table 3:** Odds ratio and 95% confidence intervals for the association between the flavonoid-rich foods and MASLD

Exposure		Low intake	Medium intake	High intake	*P* for trend
Apples and pears	Model 1	1	0.63 (0.37–1.04)	0.63 (0.39–1.04)	0.11
	Model 2	1	0.63 (0.36–1.11)	0.72 (0.41–1.27)	0.39
Grapes	Model 1	1	0.92 (0.60–1.40)	0.95 (0.59–1.54)	0.78
	Model 2	1	1.06 (0.67–1.66)	0.92 (0.54–1.57)	0.84
Oranges, mandarins, and Kiwis	Model 1	1	0.65 (0.40–1.06)	0.59 (0.38–0.91)	0.02
	Model 2	1	0.52 (0.30–0.91)	0.51 (0.31–0.85)	0.01
Bell peppers	Model 1	1	1.56 (0.99–2.44)	0.89 (0.58–1.38)	0.65
	Model 2	1	1.77 (1.08–2.89)	1.06 (0.65–1.73)	0.74
Red wine	Model 1	1	0.62 (0.37–1.03)	0.64 (0.41–1.01)	0.09
	Model 2	1	0.67 (0.38–1.19)	0.7 (0.39–1.25)	0.27
Tea	Model 1	1	1.28 (0.72–2.26)	1.02 (0.69–1.51)	0.93
	Model 2	1	0.94 (0.50–1.77)	1.12 (0.72–1.75)	0.61
Berries	Model 1	1	0.96 (0.57–1.63)	0.88 (0.53–1.47)	0.61
	Model 2	1	1.08 (0.60–1.94)	0.95 (0.53–1.69)	0.76

n = 531, values are OR (95% CI), *P* values obtained via linear trend test

Model 1 adjusted for age (years) and sex (male, premenopausal female, postmenopausal female)

Model 2 adjusted for model 1 plus BMI (≤ 25 kg/m^2^, > 25 kg/m^2^ and < 30 kg/m^2^, ≥ 30 kg/m^2^ and < 35 kg/m^2^, ≥ 35 kg/m^2^), education level (none, primary or middle; secondary; higher education), smoking status (never, < 3 months, previous, current, missing), physical activity (low, moderate, high), energy intake (KJ/day), soluble fibre intake (g/day), alcohol intake (< 8 g/day, 8–16 g/day, > 16 g/day), and coffee intake (low, moderate, high).

### Likelihood ratio tests

We observed no statistically significant interaction between major covariates and the association between the FDS and MASLD (Supplementary Table S4).

### Mediation analysis

Three microbiome variables satisfied the criteria for mediation (Table 4). Significant mediation was observed for the genus *Eisenbergiella* in the association between the FDS and MASLD (indirect effect B coefficient [95% CI], *P* value, proportion mediated (%): − 0.006 [ − 0.019 − 0.000], 0.04, 9.2%). No other variables showed any indication of statistical significance for mediation (Table 4).

**Table 4:** Mediation analysis for the association between the flavodiet score and MASLD by the microbiome

Mediator	Proportion mediated (%)	Indirect effect	*P* value
*Genus*			
*Eisenbergiella*	9.2	− 0.006 (− 0.019, − 0.000)	0.04
*Oscillibacteria*	− 0.1	0.000 (− 0.007, 0.007)	0.94
*Species*			
*Roseburia hominis*	5.8	− 0.004 (− 0.012, 0.002)	0.19
*Diversity index*			
Shannon index	1.0	− 0.001 (− 0.005, 0.004)	0.79
Simpson index	1.4	− 0.001 (− 0.006, 0.004)	0.79

n = 531

Model adjusted for age (years), sex (male, premenopausal female, postmenopausal female), BMI (≤ 25 kg/m^2^, > 25 kg/m^2^ and < 30 kg/m^2^, ≥ 30 kg/m^2^ and < 35 kg/m^2^, ≥ 35 kg/m^2^), education level (none, primary or middle; secondary; higher education), smoking status (never, < 3 months, previous, current, missing), physical activity (low, moderate, high), energy intake (KJ/day), soluble fibre intake (g/day), alcohol intake (< 8 g/day, 8-16 g/day, > 16 g/day), and coffee intake (low, moderate, high)

### Sensitivity analysis

When modelling the FDS in quartiles, the inverse association between the FDS and MASLD remained statistically significant with Q4 of the FDS being associated with 50% lower odds of MASLD when compared to Q1 (OR _Q4 vs Q1_ [95% CI], 0.50 [0.27, 0.96]; *P* value: 0.03). Additionally, a higher intake of flavanones was associated with lower odds of having MASLD when comparing Q4 to Q1 (OR _Q4 vs Q1_ [95% CI], 0.53 [0.29, 0.97]; *P* value: 0.18), although the P for trend did not reach statistical significance (Supplementary Table S5).

The exclusion of oranges, mandarins, and kiwis, and berries from the FDS attenuated the associations between the FDS and MASLD (OR [95% CI], *P* value: FDS excl. oranges, mandarins and kiwis = 0.79 [0.58, 1.07], 0.12; FDS excl. berries = 0.76 [0.56, 1.02], 0.07). (Supplementary Fig. S2). Additionally, the exclusion of each flavonoid-rich food separately in the leave-one-out sensitivity analysis attenuated the significance of the mediation by the genus *Eisenbergiella* in the association between the FDS and odds of MASLD (indirect effect B coefficient [95% CI], *P* value, proportion mediated (%): FDS excl. red wine = − 0.001 [ − 0.005, 0.000], 0.19, 6.0%; FDS excl. apple =  − 0.001 [ − 0.005, 0.000], 0.16, 5.5%; FDS excl. grapes =  − 0.001 [ − 0.005, 0.000], 0.15, 5.7%; FDS excl. berries =  − 0.001 [ − 0.004, 0.001], 0.46, 4.0%; FDS excl. oranges, mandarins and kiwis =  − 0.002 [ − 0.006, 0.000], 0.09, 9.9%; FDS excl. tea =  − 0.002 [ − 0.007, 0.000], 0.09, 8.4%; FDS excl. bell pepper =  − 0.001 [ − 0.005, 0.000], 0.14, 6.2%) (Supplementary Table S6).

## Discussion

Our data suggests that a flavonoid-rich diet comprised of higher intakes of flavonoid-rich foods including apples, oranges, and berries was associated with a 27% lower odds of having MASLD in a population-based cohort of older adults in Germany. We further observed, for the first time, that up to 9.2% of this association was mediated by a greater abundance of the Genus *Eisenbergiella*.

### Association of Flavonoids with MASLD

We have recently demonstrated in a UK-based large prospective cohort study that a flavonoid-rich diet was associated with a 19% lower risk of developing MASLD, and lower imaging-derived biomarkers of MASLD in the UK Biobank [18]. The present analyses in a moderate-sized sample from Northern Germany provides further evidence that a diet rich in flavonoids may attenuate MASLD development, while adding novel mechanistic insights into the role of the gut microbiome. Previous randomized controlled trials have reported that greater flavonoid intakes may improve short- and medium-term biomarkers of MASLD [22], while mechanistic studies have shown that a number of flavonoid compounds, including anthocyanins and flavonols, can attenuate hepatocyte dysfunction *in-vivo* and *in-vitro* [45, 46]. In population-based studies, consistent inverse associations between greater total flavonoid and flavonoid subclass intakes and MASLD have been observed, with risk reductions in prospective analyses of 19% for MASLD incidence, and 29% for MASLD progression [18–21]. Our previous analyses in the UK Biobank [18] suggest that such benefits may be achievable under real-world conditions, i.e. by a diet composed of 6 servings of common flavonoid-rich foods per day, and further, in combination with this analysis, suggest that a benefit is conferred independent of source, given we observe inverse associations with MASLD across populations where there is heterogeneity in the flavonoid-rich food sources and levels of intake.

While we observed significant associations between higher intake of the FDS and lower odds of MASLD in the present study, we only observed significant inverse associations for a limited number of specific foods, namely oranges, mandarins and kiwis. Additionally, we observed no associations with MASLD for individual flavonoid subclasses, with the exception of flavanone intake across quartiles, although there was no statistically significant trend for a linear inverse association between flavanone intake and MASLD. This may speak to the importance of the whole flavonoid-rich dietary pattern. A concept that may be further strengthened by our leave-one-out sensitivity analysis where, for the majority of exclusions, the FDS remained inversely associated with MASLD. While different flavonoid compounds may differentially affect metabolic pathways owing to their specific structure, it may be that the benefits are greatest when compound diversity is greatest, such that many metabolic pathways are simultaneously acted upon [47] which, when combined with our relatively small sample size, and low to moderate intakes of flavonoids in the PopGen sample, may explain our null findings on the food and subclass level. Alternatively, it may be some other additional component of these foods that drives these associations with MASLD such as fiber. However, our findings were adjusted for soluble fiber intake which is the main fiber source metabolised by the gut microbiome [48] and additionally, if other dietary components were responsible for driving the association of flavonoids and MASLD we would anticipate seeing significant inverse associations for most fruit and vegetable-based foods analysed, given that each food would represent intake of such a shared component.

### Potential mediating role of the gut microbiome

The role of the gut microbiome in the aetiology of MASLD has been suggested previously [49] and various flavonoid compounds have been shown to affect the composition of the gut microbiome, and the downstream metabolites that enter circulation [16]. In the present analyses, we observed that 9.2% of the association of a flavonoid-rich diet with MASLD is mediated by a greater abundance of the genus *Eisenbergiella*. Previous mechanistic work [50] has shown that the *Eisenbergiella* taxa is implicated in the conversion of rutin (a prominent dietary flavonol) to quercetin, and these compounds have been shown in cell culture and animal models to attenuate MASLD development and associated metabolic and hepatic dysfunction [51–55], with a suggestion that the benefit may be conferred by modulation of the microbiome [51]. Indeed, we did not see any statistically significant association for the flavonol subclass, of which rutin is part of, with MASLD in the present analyses. However this may be again due to the low flavonoid intakes in this sample, or due to limitations in the calculation of flavonoid intakes from grouped foods with varying concentrations of flavonoids in the FFQ. However, despite this, we see significant mediation via the taxa *Eisenbergeilla* for the association of the FDS with MASLD. Thus, the role of rutin and quercetin in MASLD development and the potential for this to be mediated by *Eisenbergeilla* should be considered as a direction for future research.

### Strengths and limitations

Strengths of the current study include the well-characterised population-based sample, the MRI-based assessment of liver fat, the detailed assessment of flavonoid intake using the EPIC FFQ, and the availability of 16S gut microbiome data for mediation analysis. Limitations to the present study include the cross-sectional design, which allows no conclusions about the temporal sequence of observed associations. Additionally, the self-reported dietary data increases the potential for recall bias to impact our associations, while grouping of flavonoid-rich foods in the dietary data also limits inference into the food-specific associations from the analysis (for example, kiwis and oranges are grouped as well as berries, thus, we cannot assess the associations for these foods separately). Furthermore, grouping may lower precision in estimating flavonoid subclass intakes from the FFQ. However, FFQ data has been shown sufficient to rank individuals according to intake, thus it is likely we can say with some accuracy that comparisons across intakes in regression models allow for hypothesis testing of higher versus lower intakes regardless of these limitations [56]. Finally, while we adjusted for a range of confounding variables in our models, we cannot rule out the possibility of residual confounding.

In conclusion, we have shown that a flavonoid-rich diet is associated with lower-odds of having MASLD in a German population, and that this association may be partially explained by a greater abundance of the Genus *Eisenbergiella* in the gut microbiome. Replication of these novel findings is the next step to further understand the relative importance of the gut microbiome in explaining the benefits of a high flavonoid diet in reducing the risk of developing MASLD.

## Supplementary Information

Below is the link to the electronic supplementary material.

Supplementary file1 (DOCX 122 kb)

## Data Availability

Data described in the manuscript, code book, and analytic code will be made available upon request pending application and approval (applications for the dataset can be submitted here: https://portal.popgen.de/).

## Abbreviations

BMI: Body mass index
EPIC: European Prospective Investigation into Cancer and Nutrition
FDS: FlavoDiet score
FFQ: Food frequency questionnaire
MRI: Magnetic resonance imaging
MASLD: Metabolic-dysfunction associated steatotic liver disease
PopGen: The population-based recruitment for genetics research
