# Supplementary material for: The gut microbiome mediates the association between a flavonoid-rich diet and MASLD in a population-level analysis
Source: Eur J Nutr. 2025 Dec 4;65(1):1. doi: 10.1007/s00394-025-03851-2 (PMC12678595; doi:10.1007/s00394-025-03851-2)

**Supplementary Material**

1. Supplementary Table S1: Variable Coding
2. Supplementary Table S2: Intakes in Categories for Flavonoid-Rich Food Variables
3. Supplementary Table S3: Mean (SD) (mg/day) Flavonoid Intakes Across Quartiles of the Flavodiet Score
4. Supplementary Table S4: Likelihood Ratio Test for Covariate Interaction
5. Supplementary Table S5: Odds Ratio and 95% Confidence Intervals for the Association between the Flavodiet Score and Flavonoid Subclasses Modelled in Quartiles, and MASLD
6. Supplementary Table S6: Leave One Out Sensitivity Analysis for Mediation by the Genus Eisenbergiella
7. Supplementary Figure S1: Flow Chart
8. Supplementary Figure S2: Leave One Out Sensitivity Analysis for the association between the FDS and MASLD

Supplementary Figure S1: Flow Chart

Total Sample (Follow-up 1)

n = 998

Missing Data (Covariates, Liver Fat, Microbiome)

n = 422

Other Liver Diseases (n = 28)

Implausible Energy (n = 0)

AST: ALT Ratio >2 (n =17)

Excessive Alcohol Intake (>60g/day for men and > 50g/day for women) (n = 16)

All Required Data

n = 576

Following Exclusions

n = 531

| Supplementary Table S1: Variable Coding | | |
| --- | --- | --- |
| Variable | Coding | Transformation |
| Covariates | | |
| Age | Continuous (years) | N/A |
| Sex | Categorical (Male, Premenopausal Female, Postmenopausal Female) | N/A |
| BMI | Categorical (≤25kg/m^2^, >25kg/m^2^ and <30kg/m^2^, ≥30kg/m^2^ and <35kg/m^2^, ≥35kg/m^2^) | N/A |
| Education | Categorical (Low, Medium, High)  Low = None, primary, or middle school  Medium = Secondary School  High = College or Higher Education | N/A |
| Smoking Status | Categorical (Never, <3 Months, Previous, Current, Missing) | N/A |
| Physical Activity | Categorical (Low, Medium, High, Missing) | N/A |
| Energy Intake | Continuous (KJ) | Natural Log |
| Soluble Fiber | Continuous (g/day) | Natural Log |
| Alcohol Intake | Categorical (quartiles) | Natural Log |
| Coffee Intake | Categorical (Low, Medium, high)  Low = 0-1  Medium = >1 – 3  High = >3 | N/A |
| Exposures | | |
| Flavodiet Score | Continuous (servings/day) (quartiles for sensitivity analysis) | Log2 |
| Flavonoid-Rich Foods | Categorical (0, <, > than median intake) | N/A |
| Flavonoid Subclasses | Continuous (mg/day) (quartiles for sensitivity analysis) | Log2 |
| Outcomes | | |
| MASLD | Categorical (No, Yes)  No = Log LSI <3  Yes = Log LSI ≥3 | N/A |
| Mediators | | |
| Microbiome Variables | All variables with >60% 0 values were excluded and analyzed as a continuous variable | Natural Log |

| Supplementary Table S2: Intakes in Categories for Flavonoid-Rich Food Variables | | | |
| --- | --- | --- | --- |
| Flavonoid-Rich Foods | Tertile 1 | Tertile 2 | Tertile 3 |
| Apples and Pears | 0 - 0.1 Servings | >0.1 – 0.50 Servings | >0.50 Servings |
| Grapes | 0 – 0.1 Servings | >0.1 – 0.21 Servings | >0.21 Servings |
| Oranges, mandarins, and kiwis | 0 – 0.1 Servings | >0.1 – 0.21 Servings | >0.21 Servings |
| Berries | 0 – 0.1 Servings | >0.1 – 0.43 Servings | >0.43 Servings |
| Red wine | 0 – 0.1 Servings | >0.1 – 0.16 Servings | >0.16 Servings |
| Tea | 0 – 0.04 Servings | >0.04 – 0.08 Servings | >0.08 Servings |
| Bell Peppers | 0 – 0.04 Servings | >0.04 – 0.08 Servings | >0.08 Servings |

| Supplementary Table S3: Mean (±SD) (mg/day) Flavonoid Intakes Across Quartiles of the FDS | | | | |
| --- | --- | --- | --- | --- |
|  | Q1 | Q2 | Q3 | Q4 |
| Total Flavonoids | 303 (184) | 609 (291) | 734 (352) | 1500 (1050) |
| Anthocyanins | 21 (17.6) | 34.5 (26.1) | 42.5 (27.1) | 55.6 (51.7) |
| Flavanones | 13.5 (17.6) | 21.5 (29.3) | 25.3 (27.6) | 28 (26.8) |
| Flavan3ols | 49.7 (46.5) | 157 (142) | 189 (159) | 570 (548) |
| Flavonols | 16 (8.9) | 25.5 (12.8) | 29.6 (13.3) | 56.9 (37.1) |
| Flavones | 3.56 (3.79) | 3.9 (2.8) | 4.61 (2.53) | 6.28 (3.92) |
| Polymers | 199 (133) | 367 (178) | 443 (211) | 785 (500) |
| Proanthocyanidins | 239 (157) | 465 (217) | 548 (259) | 1050 (655) |

| Supplementary Table S4: Likelihood Ratio Test for Covariate Interaction | |
| --- | --- |
| Covariate | P Value |
| Age | 0.84 |
| Sex | 0.48 |
| BMI | 0.47 |
| Education | 0.32 |
| Physical Activity | 0.92 |
| Smoking | 0.47 |
| Alcohol Intake | 0.10 |
| n = 531  Model adjusted for age (years), sex (male, premenopausal female, postmenopausal female), BMI (≤25kg/m^2^, >25kg/m^2^ and <30kg/m^2^, ≥30kg/m^2^ and <35kg/m^2^, ≥35kg/m^2^), education level (none, primary or middle; secondary; higher education), smoking status (never, <3 months, previous, current, missing), physical activity (low, moderate, high), energy intake (KJ/day), soluble fibre intake (g/day), alcohol intake (<8g/day, 8-16g/day, >16g/day), and coffee intake (low, moderate, high). | |

| Supplementary Table S5: Odds Ratio and 95% Confidence Intervals for the Association between the Flavodiet Score and Flavonoid Subclasses Modelled in Quartiles, and MASLD | | | | | | |
| --- | --- | --- | --- | --- | --- | --- |
| Exposure |  | Q1 | Q2 | Q3 | Q4 | P Value |
| Diet Score |  |  |  |  |  |  |
| FDS | Model 1 | 1 | 0.84 (0.50-1.40) | 0.75 (0.44-1.26) | 0.51 (0.30-0.88) | 0.02 |
|  | Model 2 | 1 | 0.82 (0.46-1.48) | 0.64 (0.35-1.19) | 0.50 (0.27-0.96) | 0.03 |
| Flavonoid Subclasses | |  |  |  |  |  |
| Anthocyanins | Model 1 | 1 | 0.70 (0.41-1.20) | 0.75 (0.44-1.28) | 0.75 (0.43-1.29) | 0.4 |
|  | Model 2 | 1 | 0.72 (0.40-1.31) | 0.82 (0.44-1.52) | 0.77 (0.40-1.49) | 0.58 |
| Proanthocyanidins | Model 1 | 1 | 1.05 (0.62-1.79) | 1.12 (0.65-1.93) | 0.88 (0.51-1.54) | 0.69 |
|  | Model 2 | 1 | 1.11 (0.61-2.02) | 1.07 (0.57-2.02) | 1.15 (0.59-2.25) | 0.74 |
| Polymers | Model 1 | 1 | 1.05 (0.62-1.78) | 0.93 (0.54-1.6) | 0.88 (0.51-1.54) | 0.57 |
|  | Model 2 | 1 | 1.17 (0.64-2.12) | 1.01 (0.54-1.9) | 1.03 (0.52-2.02) | 0.93 |
| Flavonols | Model 1 | 1 | 0.85 (0.49-1.48) | 1.22 (0.70-2.11) | 0.80 (0.45-1.43) | 0.73 |
|  | Model 2 | 1 | 0.88 (0.47-1.65) | 1.25 (0.65-2.4) | 0.93 (0.46-1.88) | 0.92 |
| Flavanones | Model 1 | 1 | 0.63 (0.37-1.05) | 1.24 (0.75-2.05) | 0.54 (0.32-0.92) | 0.19 |
|  | Model 2 | 1 | 0.66 (0.37-1.18) | 1.22 (0.68-2.18) | 0.53 (0.29-0.97) | 0.18 |
| Flavan-3-ols | Model 1 | 1 | 0.72 (0.42-1.23) | 1.06 (0.63-1.79) | 0.63 (0.37-1.09) | 0.28 |
|  | Model 2 | 1 | 0.69 (0.38-1.25) | 1.17 (0.65-2.13) | 0.69 (0.36-1.30) | 0.61 |
| Flavones | Model 1 | 1 | 1.12 (0.67-1.89) | 1.27 (0.75-2.13) | 0.90 (0.52-1.53) | 0.79 |
|  | Model 2 | 1 | 1.27 (0.70-2.29) | 1.40 (0.76-2.58) | 0.98 (0.52-1.84) | 0.93 |
| n = 531, values are OR (95% CI), P Values obtained via linear trend test.  Model 1 adjusted for age (years) and sex (male, premenopausal female, postmenopausal female)  Model 2 adjusted for model 1 plus BMI (≤25kg/m2, >25kg/m2 and <30kg/m2, ≥30kg/m2 and <35kg/m2, ≥35kg/m2), education level (none, primary or middle; secondary; higher education), smoking status (never, <3 months, previous, current, missing), physical activity (low, moderate, high), energy intake (KJ/day), soluble fibre intake (g/day), alcohol intake (<8g/day, 8-16g/day, >16g/day), and coffee intake (low, moderate, high). | | | | | | |

| Supplementary Table S6: Leave One Out Sensitivity Analysis for Mediation by the Genus Eisenbergiella | | | |
| --- | --- | --- | --- |
|  | Proportion Mediated (%) | Indirect Effect | P Value |
| FDS excl. Red Wine | 6.0 | -0.001 (-0.004, 0.001) | 0.21 |
| FDS excl. Apple | 5.5 | -0.001 (-0.004, 0.000) | 0.17 |
| FDS excl. Grapes | 5.7 | -0.001 (-0.004, 0.000) | 0.16 |
| FDS excl. Berries | 4.0 | -0.001 (-0.003, 0.001) | 0.45 |
| FDS excl. Oranges | 9.9 | -0.002 (-0.005, -0.000) | 0.10 |
| FDS excl. Tea | 8.4 | -0.002 (-0.006, -0.000) | 0.08 |
| FDS excl. Bell Pepper | 6.2 | -0.001 (-0.004, 0.000) | 0.13 |
| n = 531, values are proportion mediated (%), the indirect effect (95% CI), and P value.  Model is adjusted for age (years), sex (male, premenopausal female, postmenopausal female), BMI (≤25kg/m2, >25kg/m2 and <30kg/m2, ≥30kg/m2 and <35kg/m2, ≥35kg/m2), education level (none, primary or middle; secondary; higher education), smoking status (never, <3 months, previous, current, missing), physical activity (low, moderate, high), energy intake (KJ/day), soluble fibre intake (g/day), alcohol intake (<8g/day, 8-16g/day, >16g/day), and coffee intake (low, moderate, high). | | | |

Supplementary Figure S2: Leave One Out Sensitivity Analysis for the association between the FDS and MASLD

n = 531, values are OR (95% CI).

Model adjusted for age (years) , sex (male, premenopausal female, postmenopausal female), BMI (≤25kg/m2, >25kg/m2 and <30kg/m2, ≥30kg/m2 and <35kg/m2, ≥35kg/m2), education level (none, primary or middle; secondary; higher education), smoking status (never, <3 months, previous, current, missing), physical activity (low, moderate, high), energy intake (KJ/day), soluble fibre intake (g/day), alcohol intake (<8g/day, 8-16g/day, >16g/day), and coffee intake (low, moderate, high).

**Exposure OR (95% CI)**


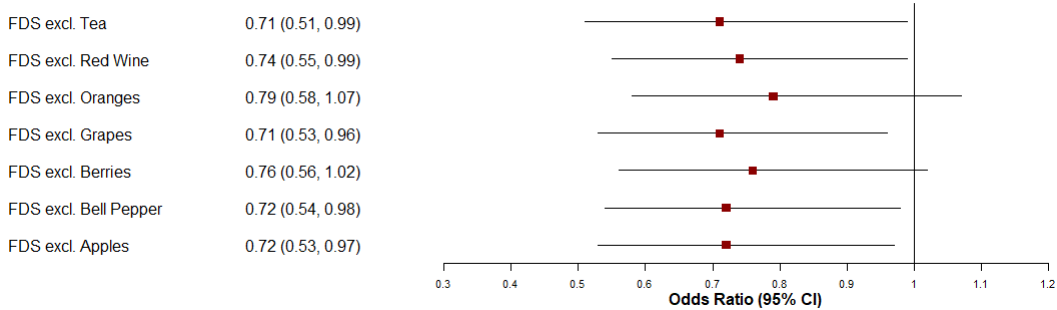

Supplement: Supplementary file 1 — Supplementary file1 (DOCX 122 kb) [file 394_2025_3851_MOESM1_ESM.docx]
